# Clinical simulation for nursing competence development in cardiopulmonary resuscitation: systematic review

**DOI:** 10.1590/1518-8345.4094.3391

**Published:** 2020-11-06

**Authors:** Juliana da Silva Garcia Nascimento, Kleiton Gonçalves do Nascimento, Jordana Luiza Gouvêa de Oliveira, Mateus Goulart Alves, Aline Roberta da Silva, Maria Celia Barcellos Dalri

**Affiliations:** 1 Universidade de São Paulo Escola de Enfermagem de Ribeirão Preto, PAHO/WHO Collaborating Centre for Nursing Research Development, Ribeirão Preto, SP, Brazil.; 2 Scholarship holder at the Coordenação de Aperfeiçoamento de Pessoal de Nível Superior (CAPES), Brazil.; 3 Universidade Federal do Triângulo Mineiro, Uberaba, MG, Brazil.; 4 Universidade de Franca, Franca, SP, Brazil.

**Keywords:** Students, Nursing, Simulation Technique, Teaching, Clinical Competence, Cardiopulmonary Resuscitation, Learning, Estudantes de Enfermagem, Simulação, Ensino, Competência Clínica, Ressuscitação Cardiopulmonar, Aprendizagem, Estudiantes de Enfermería, Simulación, Enseñanza, Competencia Clínica, Reanimación Cardiopulmonar, Aprendizaje

## Abstract

**Objective:**

to identify the effectiveness of clinical simulation for competence development regarding cardiopulmonary resuscitation in comparison with different teaching and learning strategies used in the education of nursing students.

**Method:**

systematic review, performed on the databases PubMed^®^/MEDLINE^®^, LILACS, Scopus, CINAHL and Web of Science. The Rayyan QCRI application was used to select the studies, in addition to the instruments for assessing the methodological quality of Joanna Briggs Institute and the Medical Education Research Study Quality Instrument.

**Results:**

a total of 887 studies were identified, and five we included in the final sample. The included studies had good methodological quality by the assessment instruments. All of them had statistically significant results to develop competence through clinical simulation, when compared to other methods.

**Conclusion:**

clinical simulation proved to be effective for the development of clinical competence in cardiopulmonary resuscitation of nursing students.

## Abstract (pt)

**Objetivo:**

identificar a efetividade da simulação clínica para desenvolvimento de competência quanto à ressuscitação cardiopulmonar em comparação com diferentes estratégias de ensino e aprendizagem utilizadas na educação de graduandos em enfermagem.

**Método:**

revisão sistemática, realizada nas bases PubMed^®^/MEDLINE^®^, LILACS, Scopus, CINAHL e *Web of Science*. Utilizou-se o aplicativo *Rayyan* QCRI para seleção dos estudos, além dos instrumentos de avaliação da qualidade metodológica do Instituto Joanna Briggs e o *Medical Education Research Study Quality Instrument*.

**Resultados:**

identificaram-se 887 estudos, sendo que cinco compuseram a amostra final. Os estudos incluídos foram considerados de boa qualidade metodológica pelos instrumentos de avaliação. Todos apresentaram resultados estatisticamente mais significativos para desenvolver competência por meio da simulação clínica, quando comparados a outros métodos.

**Conclusão:**

a simulação clínica mostrou-se efetiva para o desenvolvimento de competência clínica na ressuscitação cardiopulmonar de estudantes em enfermagem.

## Abstract (es)

**Objetivo:**

identificar la eficacia de la simulación clínica para desarrollar competencias de la resucitación cardiopulmonar en comparación con diferentes estrategias de enseñanza y aprendizaje que se utilizan en la educación de estudiantes de enfermería.

**Método:**

se trata de una revisión sistemática, realizada en las bases PubMed^®^/MEDLINE^®^, LILACS, Scopus, CINAHL y *Web of Science*. Se utilizó la aplicación *Rayyan* QCRI para seleccionar los estudios, además de los instrumentos de evaluación de la calidad metodológica del Instituto Joanna Briggs y el *Medical Education Research Study Quality Instrument*.

**Resultados:**

se identificaron 887 estudios de los cuales 5 compusieron la muestra final. Los instrumentos de evaluación reconocieron a los estudios incluidos como de buena calidad metodológica. Todos presentaron mejores resultados estadísticamente significativos para desarrollar competencias por medio de la simulación clínica, al ser comparados con otros métodos.

**Conclusión:**

la simulación clínica ha resultado eficaz para el desarrollo de competencias clínicas en la resucitación cardiopulmonar de los estudiantes de enfermería.

## Introduction

Adopting new teaching and learning strategies in nursing is very important for excellence in the development of students’ knowledge, skills and attitudes^(^ 1 ^-^ 2 ^)^. Thus, clinical simulation, configured as a pedagogical mechanism for teaching and learning in health, which imitates real clinical care, has gained space in nursing education, characterized as an experiential, interactive, collaborative and learner-centered strategy^(^ 3 ^)^.

Specifically regarding teaching and learning of cardiopulmonary resuscitation (CPR) for nursing, strategies frequently adopted by educators are still guided by traditional approaches, such as lectures supported by PowerPoint^®^ presentations and laboratory skills training guided by an instructor^(^ 4 ^-^ 5 ^)^.

This classic pattern of CPR training has shown ineffective results for care quality, such as a decrease in the cognitive and psychomotor skills of individuals 1 month after the completion of the courses^(^ 6 ^-^ 7 ^)^. However, it is not yet clear whether new teaching and learning strategies, such as clinical simulation, are more effective in developing the competence of nursing students to attend CPR^(^ 4 ^,^ 8 ^)^.

The evaluation of the development of clinical competence, defined as the application of skills in all domains of practice, articulating knowledge, skills and attitudes in different clinical contexts^(^ 7 ^,^ 9 ^)^, is considered a complex and difficult to handle topic. Its use in the teaching of CPR to nursing students was verified in studies whose outcomes were varied and not always conclusive regarding its effectiveness^(^ 10 ^-^ 12 ^)^.

This study aimed to identify the effectiveness of clinical simulation for competence development regarding CPR in comparison with different teaching and learning strategies used in the education of nursing students.

## Method

This is a systematic literature review, prepared in accordance with the Preferred Reporting Items for Systematic Reviews and Meta-Analysis (PRISMA) strategy^(^ 13 ^)^, from July to October 2019.

To comply with this systematic review, seven steps were followed: (1) definition of the research question, specifying the population and the intervention of interest; (2) identification of databases, descriptors, keywords and search strategies; (3) establishment of inclusion and exclusion criteria; (4) search databases with up to two independent researchers; (5) comparison of examiners’ searches and definition of initial study selection; (6) application of the inclusion criteria and justification for possible exclusions, along with the critical analysis of all studies included in the review; (7) elaboration of a critical summary, synthesizing the information made available by the articles included in the review, and presentation of conclusion, informing the evidence on the effects of the intervention^(^ 14 ^)^.

The research question was defined through the Patient - Intervention - Comparison-Outcomes (PICO) strategy^(^ 15 ^)^ with the following elements: the acronym P referred to undergraduate nursing students; I, clinical simulation; C, different teaching and learning strategies; and O, the development of clinical competence for CPR. Thus, the following guiding question was structured: What is the effectiveness of clinical simulation in comparison with different teaching and learning strategies for competence development regarding CPR in nursing students?

The following databases were defined as data source: PubMed^®^/MEDLINE^®^, Latin American and Caribbean Literature on Health Sciences (LILACS), Scopus, Cumulative Index to Nursing and Allied Health Literature (CINAHL) and Web of Science.

According to the database, specific descriptors and search strategies were used. In PubMed^®^ and Scopus, the descriptors found in Medical Subjects Headings (MESH) “Students, Nursing”, “Simulation Training”, “Teaching”, “Clinical Competence” and “Cardiopulmonary Resuscitation” were used, and the search strategies were P versus I − (“Students, Nursing” OR “Pupil Nurses” OR “Student, Nursing” OR “Nurses, Pupil” OR “Nurse, Pupil” OR “Pupil Nurse” OR “Nursing Student” OR “Nursing Students”) AND (“Training, Simulation” OR “Interactive Learning” OR “Learning, Interactive”) - and I versus C versus O − (“Training, Simulation” OR “Interactive Learning” OR “Learning, Interactive”) AND (Teaching OR “Training Techniques” OR “Technique, Training” OR “Techniques, Training” OR “Training Technique” OR “Training Technics” OR “Technic, Training” OR “Technics, Training” OR “Training Technic” OR “Pedagogy” OR “Pedagogies” OR “Teaching Methods” OR “Method, Teaching” OR “Methods, Teaching” OR “Teaching Method” OR “Academic Training” OR “Training, Academic” OR “Training Activities” OR “Activities, Training” OR “Training Activity” OR “Techniques, Educational” OR “Technics, Educational” OR “Educational Technics” OR “Educational Technic” OR “Technic, Educational” OR “Educational Techniques” OR “Educational Technique” OR “Technique, Educational”) AND (“Clinical Competence” OR “Competency, Clinical” OR “Competence, Clinical” OR “Clinical Competency” OR “Clinical Competencies” OR “Competencies, Clinical” OR “Clinical Skill” OR “Skill, Clinical” OR “Skills, Clinical” OR “Clinical Skills”) AND (“Clinical Competence” OR “Competency, Clinical” OR “Competence, Clinical” OR “Clinical Competency” OR “Clinical Competencies” OR “Competencies, Clinical” OR “Clinical Skill” OR “Skill, Clinical” OR “Skills, Clinical” OR “Clinical Skills”) AND (“Cardiopulmonary Resuscitation” OR “Resuscitation, Cardiopulmonary” OR CPR OR “Cardio-Pulmonary Resuscitation” OR “Cardio Pulmonary Resuscitation” OR “Resuscitation, Cardio-Pulmonary” OR “Code Blue” OR “Mouth-to-Mouth Resuscitation” OR “Mouth to Mouth Resuscitation” OR “Mouth-to-Mouth Resuscitations” OR “Resuscitation, Mouth-to-Mouth” OR “Resuscitations, Mouth-to-Mouth” OR “Basic Cardiac Life Support” OR “Life Support, Basic Cardiac”).

In CINAHL, the descriptors were “Students, Nursing”, “Simulations”, “Teaching”, “Clinical Competence” and “Resuscitation, Cardiopulmonary”, identified in titles, and the following search strategies were applied: P versus I - (“Students, Nursing”) AND (Simulations) - and I versus C versus O - (Simulations) AND (Teaching OR “Models, Educational”) AND (“Clinical Competence”) AND (“Resuscitation, Cardiopulmonary”).

In Web of Science, the descriptors “Students, Nursing”, “Simulation Training”, “Teaching”, “Clinical Competence” and “Cardiopulmonary Resuscitation” were used. The search strategy was configured as: (“Students, Nursing” AND “Simulation Training” AND Teaching AND “Clinical Competence” AND “Cardiopulmonary Resuscitation”).

At LILACS, the following Health Science Descriptors (DeCS) were searched: “Nursing Students”, “Simulation Training”, “Teaching”, “Clinical Competence”, “Cardiopulmonary Resuscitation” and their English and Spanish versions, with the following search strategy: (“Students, Nursing”) AND (“Simulation Training”) AND (Teaching) AND (“Clinical Competence”) AND (“Cardiopulmonary Resuscitation”) (“Estudiantes de Enfermería”) AND (“Entrenamiento Simulado”) AND (Enseñanza) AND (“Competencia Clínica”) AND (“Reanimación Cardiopulmonar”) (“Nursing Students”) AND (“Simulation Training”) AND (Teaching) AND (“Clinical Competence”) AND (“Cardiopulmonary Resuscitation”).

Primary studies of clinical trial type, randomized or not, were included, which presented the comparison of the effectiveness of clinical simulation to develop competence on CPR in adults with other teaching and learning strategies applied to undergraduate nursing students, without delimited timeline; published in Portuguese, English and Spanish; in scientific journals and electronically available. Studies that addressed nursing professionals, neonatal and pediatric CPR, literature reviews, editorials, reviews, experience reports, case studies, theoretical reflections, dissertations, theses, monographs and summaries published in annals of events were excluded.

The studies were identified in the information sources selected by two independent researchers, previously trained to evaluate titles and abstracts, through a single version free web review program named Rayyan Qatar Computing Research Institute (Rayyan QCRI)^(^ 16 ^)^, identified at the link https://rayyan.qcri.org/.

Rayyan QCRI helps authors of systematic reviews to carry out their work quickly, easily and pleasantly, allowing the export of studies from a certain database for the program and the exposure of titles and abstracts, with the blindness of the auxiliary researcher, which guarantees reliability in the selection of information, accuracy and methodological precision^(^ 16 ^)^.

The 12 studies that showed divergence were sent to a third researcher, specialized in the theme, responsible for making the decision to include or exclude, and then a critical analysis of the articles was carried out. After we observed the incipience of selected studies, the references of the included articles were analyzed, without resulting in new additions to the final sample.

In data collection, the criteria from a validated instrument^(^ 17 ^)^ were used, addressing title, authors, year of publication, origin of the study, language, journal, objectives, methodological design, results and conclusion. The Evidence Level^(^ 18 ^)^ was also classified and the selection and inclusion of studies was demonstrated following the recommendations of the Preferred Reporting Items for Systematic Review and Meta-Analyzes-PRISMA^(^ 13 ^)^.

The methodological evaluation of the selected studies was carried out according to the critical evaluation instruments from Joanna Briggs Institute^(^ 19 ^)^ and Medical Education Research Study Quality Instrument (MERSQI)^(^ 20 ^)^. We opted to use both to obtain a broad scenario of evaluation of the articles methodological quality, since the adopted instruments have different perspectives and evaluation criteria.

The instrument referring to Joanna Briggs Institute has a total of nine items of methodological evaluation aimed at quasi-experimental studies and 13 for experimental ones and considers whether they are present, absent, and whether there is clarity or not^(^ 19 ^)^. MERSQI consists of a total of six domains, composed of criteria that assess the methodological quality of the studies: (1) study design (only one group or a post-test, 1 point; pre-test and post-test of a single group, 1.5 points, two non-randomized groups, 2 points and a randomized study, 3 points); (2) sample (one studied institution, 0.5 point; two institutions, 1 point; three studied institutions, 1.5 point and the sample response rate <50%, 0.5 point; 50% to 74%, 1 point and >75%, 1.5 points); (3) data type (assessment made by the participants, 1 point and objective assessment, 2 points); (4) validity of the assessment instrument (internal structure not reported, zero point; reported, 1 point; unreported content, zero point; reported content, 1 point; relations with other unreported variables, zero point and reported relations, 1 point); (5) data analysis (inappropriate for the study design or data type, zero point; appropriate for the study design, 1 point; only descriptive analysis, 1 point; in addition to descriptive analysis, 2 points); and (6) results (obtaining knowledge and skills, 1.5 points; satisfaction, attitudes, perceptions, opinions, general facts and confidence, 1 point). The maximum score is 18^(^ 20 ^)^. Studies with scores ≤10 are considered low quality ones; from >10 to <15, moderate quality; and ≥15, high quality^(^ 21 ^)^.

## Results

**Figure 1:**
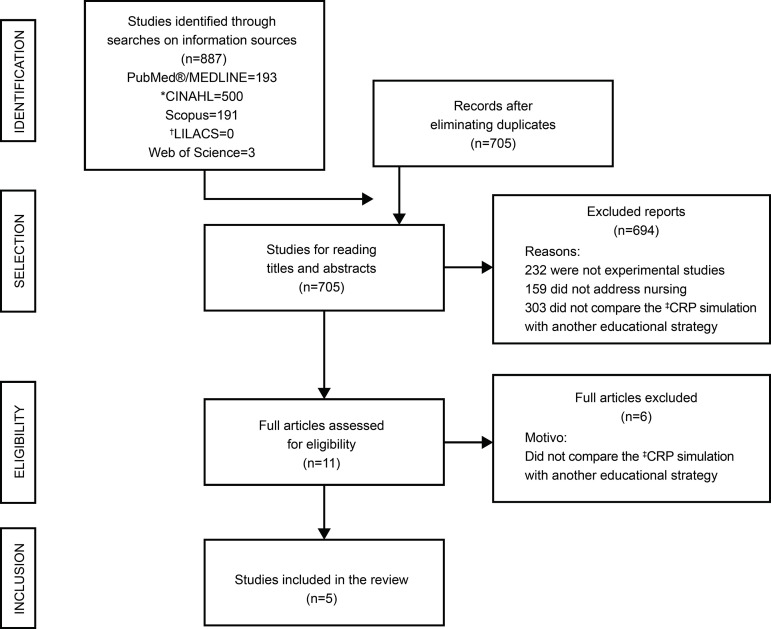
Flowchart of the identification, selection and inclusion process of studies adapted from the Preferred Reporting Items for Systematic Review and Meta-Analyzes (PRISMA). Despite the adoption of the instrument for critical evaluation of the studies from Joanna Briggs Institute, a protocol was not registered for this systematic review^1^. Ribeirão Preto, SP, Brazil, 2019 *CINAHL = Cumulative Index to Nursing and Allied Health Literature; †LILACS = Latin American and Caribbean Literature in Health Sciences; ‡CPR = Cardiopulmonary resuscitation

Source: Moher, et al.^(^13^)^

The selection and inclusion of the studies in this research is shown in Figure 1, following the recommendations of the Preferred Reporting Items for Systematic Review and Meta-Analyzes-PRISMA^(^ 13 ^).^

Figure 2 shows the critical evaluation of the methodological quality of quasi-experimental studies, according to the evaluation instrument from Joanna Briggs Institute^(^ 19 ^)^.

**Figure 2:** Evaluation of quasi-experimental studies included in the review, according to Joanna Briggs Institute methodological quality assessment instrument. Ribeirão Preto, SP, Brazil, 2019

Questions	Bruce, et al.^(^ 22 ^)^	Ackermann^(^ 23 ^)^	Akhu-Zaheya, et al.^(^ 24 ^)^
1. Is it clear in the study what the “cause” and “effect” are, that is, is there no confusion which variable comes first?	Yes	Yes	Yes
2. Do the participants included in the groups have similar characteristics for comparison?	Yes	Yes	Yes
3. Did the participants included in the groups receive similar treatment in the intervention of interest?	Yes	Yes	Yes
4. Was there a control group?	Yes	Yes	Yes
5. Were there multiple measurements of the pre- and post-intervention/exposure outcome over time?	Yes	No	No
6. Was the follow-up complete and, if not, were the differences between the groups properly described and analyzed?	Yes	Yes	Yes
7. Were the participants’ results, in any comparisons, measured in the same way?	Yes	Yes	Yes
8. Were results measured reliably?	Yes	Yes	Yes
9. Were appropriate statistical analyzes used?	Yes	Yes	Yes

Figure 3 shows the critical evaluation of the methodological quality of experimental studies, according to Joanna Briggs Institute evaluation instrument^(^ 19 ^)^.

**Figure 3:** Evaluation of the methodological quality of experimental studies included in the review, according to Joanna Briggs Institute critical evaluation instrument. Ribeirão Preto, SP, Brazil, 2019

Questions	Aqel, et al.^(^ 25 ^)^	Tawalbeh, et al.^(^ 26 ^)^
1. Was the randomization used to allocate participants to treatment groups?	Yes	Yes
2. Was the researcher responsible for allocation to the treatment groups blinded?	It is not clear	It is not clear
3. Were the treatment groups similar?	Yes	Yes
4. Were the participants blinded in allocating treatment?	It is not clear	It is not clear
5. Were those responsible for providing treatment blinded?	It is not clear	It is not clear
6. Were the outcome assessors blinded regarding the allocation of treatment?	It is not clear	It is not clear
7. Were the treatment groups treated in the same way as the intervention of interest?	Yes	Yes
8. Was the follow-up completed and, if not, were the differences between the groups in terms of follow-up properly described and analyzed?	Yes	Yes
9. Were the participants analyzed in the groups to which they were allocated?	Yes	Yes
10. Were the results measured in the same way for treatment groups?	Yes	Yes
11. Were the results measured reliably?	Yes	Yes
12. Was appropriate statistical analysis used?	Yes	Yes
13. Was the study design appropriate, and was there any deviation from the standard RCT* design in conducting and analyzing?	Yes	Yes

* RCT = Randomized clinical trial

The quasi-experimental studies included in this review met most of the quality assessment requirements indicated by the instrument of Joanna Briggs Institute, being considered of good quality. Only the criterion that addresses the use of multiple measurements of results in pre- and post-intervention/exposure over time has not been met in two studies^(^ 23 ^-^ 24 ^)^.

As for experimental studies, despite the fact that most criteria indicated for quality assessment have been met, there was a significant methodological weakness regarding the blinding of the researcher, participants, those responsible for providing treatment and results evaluators regarding the allocation of treatment.

MERSQI was also used to assess the methodological quality of the studies included in the sample, shown in Figure 4.

**Figure 4:** Evaluation of the methodological quality of the studies, according to Medical Education Research Study Quality Instrument. Ribeirão Preto, SP, Brazil, 2019

Domains	Bruce, et al.^(^ 22 ^)^	Ackermann^(^ 23 ^)^	Akhu-Zaheya, et al.^(^ 24 ^)^	Aqel, et al.^(^ 25 ^)^	Tawalbeh, et al.^(^ 26 ^)^
Study design	Non-randomized: 2 points	Non-randomized: 2 points	Non-randomized: 2 points	Randomized study: 3 points	Randomized study: 3 points
Sample (number of centers where the study was conducted and response rate)	A single institution: 0.5 point 50%-74% response rate: 1 point	A single institution: 0.5 point >75% response rate: 1.5 point	A single institution: 0.5 point 50%-74% response rate: 1 point	A single institution: 0.5 point >75% response rate: 1.5 point	A single institution: 0.5 point >75% response rate: 1.5 point
Data type/evaluation	Subjective evaluation: 1 point Objective evaluation: 2.0	Objective evaluation: 2.0 points	Objective evaluation: 2.0 points	Objective evaluation: 2.0 points	Objective evaluation: 2.0 points
Validity of the evaluation instrument	Internal structure, content, relations with other unreported variables: 0 point	Internal structure and reported content: 1 point Relations with other unreported variables:0	Internal structure and reported content: 1 point Relations with other unreported variables:0	Internal structure, content, relations with other unreported variables: 0 point	Internal structure and reported content: 1 point Relations with other unreported variables:0
Data analysis	Appropriate for study design: 1 point In addition to the descriptive analysis: 2 points	Appropriate for study design: 1 point In addition to the descriptive analysis: 2 points	Appropriate for study design: 1 point In addition to the descriptive analysis: 2 points	Appropriate for study design: 1 point In addition to the descriptive analysis: 2 points	Appropriate for study design: 1 point In addition to the descriptive analysis: 2 points
Results	Knowledge and skills: 1.5 points Satisfaction, attitudes, perceptions, opinions, general facts and confidence: 1 point	Knowledge and skills: 1.5 points	Knowledge and skills: 1.5 points Satisfaction, attitudes, perceptions and confidence: 1 point	Knowledge and skills: 1.5 points	Knowledge and skills: 1.5 points Satisfaction, attitudes, perceptions and confidence: 1 point
Total score	11.0	12.5	13	11.5	14.5

Through the use of MERSQI, it is possible to state that the studies included in the sample of this review had moderate methodological quality (10< n <15), with an average score of 12.5 points, a minimum value of 11 and a maximum value of 14.5 points. The criteria responsible for conferring methodological weakness were the execution of the studies in a single center/institution and the lack of clarification as to the validity of the assessment instruments highlighted by these manuscripts.

The articles included in the review are summarized in Figure 5. All of them were international publications. The authors identified that clinical simulation was an effective teaching and learning strategy to develop clinical competence in nursing students for cardiopulmonary resuscitation, when compared to other teaching mechanisms.

**Figure 5:** Characterization of the studies that comprised the sample of this systematic review. Ribeirão Preto, SP, Brazil, 2019

Author, year and country	Objectives	Method	Results/conclusion	Evidence level
Bruce, et al.^(^ 22 ^)^ 2009, United States of America	To compare the effectiveness of clinical laboratory simulation and a virtual computer game, regarding the development of competence for CPR* in nursing students	Quasi-experiment. Undergraduate nursing course at an American university. The control group had clinical simulation on-site; the experimental group had a virtual computer game for CPR	The scores for cognitive knowledge were significant in both teaching strategies (p=0.000), while post-simulation confidence scores were not statistically significant (p=0.177). The use of virtual simulation in CPR is effective, but on-site simulation is necessary to develop confidence in students	3
Ackermann^(^ 23 ^)^ 2009, United States of America	To compare the effectiveness of clinical CPR simulation for nursing students with a traditional CPR teaching strategy	Quasi-experiment. Undergraduate nursing course (65 American students). The experimental group had classes, skills training and clinical simulation in CPR; the control group had classes and skills training	The experimental group proved to be statistically more significant in the development of clinical competence in CPR when compared to the traditional strategy. Teaching CPR through clinical simulation is effective for nursing	3
Akhu-Zaheya, et al.^(^ 24 ^)^, 2013, Jordan	To examine the effectiveness of clinical simulation for CPR regarding knowledge acquisition, retention and self-efficacy of Jordanian nursing students	Quasi-experiment. Undergraduate nursing course in Jordan (110 students). The experimental group (n=52) had PowerPoint classes, skills training and clinical simulation, while the control group (n=58) had PowerPoint classes and skills training	The experimental group achieved higher scores for knowledge acquired and retained in CPR and greater perception of self-efficacy. Nursing students should be educated with more realistic technologies, such as simulation	3
Aqel, et al.^(^ 25 ^)^, 2014, Jordan	To examine the effectiveness of clinical CPR simulation for competence development and retention in nursing students	Randomized experiment. Undergraduate nursing course in Jordan (90 students). The experimental group had PowerPoint classes and clinical CPR simulation, and the control group had PowerPoint classes and CPR skills training	Significant differences were identified in favor of the experimental group in the development of competence for CPR. The results of this study favor the use of simulation for nursing education	2
Tawalbeh, et al.^(^ 26 ^)^, 2014, Jordan	To examine the effect of clinical simulation on cognitive knowledge, knowledge retention and nursing students’ confidence in CPR	Randomized experiment (100 students). Undergraduate nursing course in Jordan. The experimental group (n=50) had CPR simulation, PowerPoint presentation and skills training. The control group had PowerPoint classes and CPR skills training	The experimental group had greater knowledge about CPR and confidence, compared to the control group. Simulation is significantly more effective than traditional training for teaching nursing students	2

* CPR = Cardiopulmonary resuscitation

## Discussion

A total of three quasi-experimental studies^(^ 22 ^-^ 24 ^)^ and two experimental^(^ 25 ^-^ 26 ^)^ were included in the sample of this review. Although all authors have pointed to clinical simulation as an effective strategy to develop clinical competence for cardiopulmonary resuscitation in nursing students, the scarcity of identified studies demonstrates the need for further scientific exploration in this area^(^ 9 ^)^.

Most^(^ 22 ^-^ 23 ^,^ 25 ^-^ 26 ^)^ studies have compared clinical simulation in CPR with traditional teaching and learning strategies for nursing, such as lecture with the support of PowerPoint presentations and the training of laboratory skills using a low fidelity manikin. They obtained statistically significant results for clinical simulation in CPR in view of other exposed methods, which can subsidize their use in nursing education^(^ 9 ^,^ 22 ^-^ 23 ^)^.

A randomized experimental study carried out with a total of 31 Chinese nurses, that compared the effectiveness of simulation to develop nursing competence with traditional teaching strategies corroborates this statements, highlighting statistically significant results for the increase of cognitive and psychomotor skills (p=0.001), a reduction in stress levels (p=0.011) and increased confidence (p=0.026)^(^ 27 ^)^.

Other studies have also pointed out clinical simulation as an innovative pedagogical strategy for the development of cognitive, psychomotor and affective skills in nursing, affirming its effectiveness for the development of clinical competence^(^ 25 ^,^ 28 ^-^ 30 ^)^.

This review used two different instruments^(^ 19 ^-^ 20 ^)^ to assess the methodological quality of the selected article sample. Joanna Briggs Institute instrument pointed out the good quality of quasi-experimental and experimental studies, but it highlighted the blinding criterion as an important methodological weakness in experimental studies.

The absence of the participant, personnel or statistician blinding, in a scientific study, can compromise the methodological quality by providing biased behavior of those involved and research bias, which affects the internal validity of the investigations and makes the effectiveness of the research uncertain. However, it is worth noting the existing difficulty to perform blinding in educational experiments, mainly due to the impossibility of guaranteeing the absence of exchange of information among the participants involved^(^ 19 ^,^ 31 ^-^ 36 ^)^.

MERSQI, another instrument used in this study, is described as reliable because it provides accuracy in identifying the methodological quality of the articles^(^ 37 ^)^. This assessment instrument identified a moderate level of quality in the researches that comprised the sample, highlighting as main weaknesses the execution of studies in a single center/institution and the lack of clarification of the validity of the instruments used.

This result is similar to a systematic review study on education based on simulation in nursing, which also used MERSQI and indicated moderate methodological quality for a sample of a total of 26 articles, in addition to the need for improvement in the preparation of educational intervention studies in nursing, mainly because they are performed in a single center and do not consider the previous validation of the instruments used^(^ 37 ^)^.

Despite the fact that multicenter clinical trials are considered the gold standard in research, since they attend different communities and reduce the time of experimentation, they are generally expensive and complex studies, which makes its execution difficult and can justify the fact that most scientific publications on pedagogical interventions in nursing be carried out in a single center^(^ 38 ^)^.

On the other hand, the validation of instruments is a criterion of fundamental methodological quality, as it confers the reliability of the intended findings. The lack of clarification on this issue, in the studies that comprised the sample of this review, may compromise the reliability of the statistical conclusions of researches and justify its moderate methodological quality^(^ 39 ^)^.

This study had two main limitations: the incipience of quasi-experimental and experimental articles on the effectiveness of simulation to develop clinical competence in CPR; and the difficulty to compare the results of the studies that made up the sample, in view of the approach to different evaluation instruments.

Based on the findings of this systematic review, it is possible to add scientific evidence capable of supporting the teaching and learning process of CPR in undergraduate nursing through clinical simulation, indicating its effectiveness, focusing on the methodological quality of studies, which is an important resource in view of the accelerated growth of information.

## Conclusion

We identified a total of five studies that confirm the effectiveness of clinical simulation to develop competence in cardiopulmonary resuscitation for nursing, in view of other pedagogical strategies. Joanna Briggs Institute methodological assessment instrument demonstrated good quality of the included studies, and the Medical Education Research Study Quality Instrument showed moderate methodological quality.

This study contributes to teaching, research and nursing care, as it demonstrates the effectiveness of simulation as a teaching and learning strategy, indicating it as a pedagogical possibility to develop clinical competence in cardiopulmonary resuscitation.

## Footnotes

1 It is noteworthy that, despite the adoption of the instrument for critical evaluation of the studies from Joanna Briggs Institute, a protocol was not registered for this systematic review. Own financing was obtained and there were no conflicts of interest.
